# The Flt3-inhibitor quizartinib augments apoptosis and promotes maladaptive remodeling after myocardial infarction in mice

**DOI:** 10.1007/s10495-023-01911-8

**Published:** 2023-11-09

**Authors:** Daria Monogiou Belik, Riccardo Bernasconi, Lifen Xu, Giacomo Della Verde, Vera Lorenz, Vivienne Grüterich, Melania Balzarolo, Michika Mochizuki, Otmar Pfister, Gabriela M. Kuster

**Affiliations:** 1 Department of Biomedicine, University Hospital Basel and University of Basel, Hebelstrasse 20, Basel, 4031 Switzerland; 2 Department of Cardiology, University Heart Center, University Hospital Basel, Basel, Switzerland

**Keywords:** Cardiotoxicity, Tyrosine kinase inhibitors, Fms-like tyrosine kinase 3, Quizartinib, Remodeling

## Abstract

**Background:**

Tyrosine kinase inhibitors (TKIs) targeting fms-like tyrosine kinase 3 (Flt3) such as quizartinib were specifically designed for acute myeloid leukemia treatment, but also multi-targeting TKIs applied to solid tumor patients inhibit Flt3. Flt3 is expressed in the heart and its activation is cytoprotective in myocardial infarction (MI) in mice.

**Objectives:**

We sought to test whether Flt3-targeting TKI treatment aggravates cardiac injury after MI.

**Methods and results:**

Compared to vehicle, quizartinib (10 mg/kg/day, gavage) did not alter cardiac dimensions or function in healthy mice after four weeks of therapy. Pretreated mice were randomly assigned to MI or sham surgery while receiving quizartinib or vehicle for one more week. Quizartinib did not aggravate the decline in ejection fraction, but significantly enhanced ventricular dilatation one week after infarction. In addition, apoptotic cell death was significantly increased in the myocardium of quizartinib-treated compared to vehicle-treated mice. In vitro, quizartinib dose-dependently decreased cell viability in neonatal rat ventricular myocytes and in H9c2 cells, and increased apoptosis as assessed in the latter. Together with H_2_O_2,_ quizartinib potentiated the phosphorylation of the pro-apoptotic mitogen activated protein kinase p38 and augmented H_2_O_2_-induced cell death and apoptosis beyond additive degree. Pretreatment with a p38 inhibitor abolished apoptosis under quizartinib and H_2_O_2_.

**Conclusion:**

Quizartinib potentiates apoptosis and promotes maladaptive remodeling after MI in mice at least in part via a p38-dependent mechanism. These findings are consistent with the multi-hit hypothesis of cardiotoxicity and make cardiac monitoring in patients with ischemic heart disease under Flt3- or multi-targeting TKIs advisable.

**Supplementary Information:**

The online version contains supplementary material available at 10.1007/s10495-023-01911-8.

## Introduction

Improvements in cancer therapy have impressively increased the lifespan of cancer patients. However, cardiovascular complications remain a primary and potentially life-limiting side effect of cancer treatment [40]. Cardiomyopathy and heart failure are among the most severe manifestations of cardiotoxicity [26]. In addition to anthracyclines, they can also be observed under targeted therapies, including tyrosine kinase inhibitors (TKIs) [2].

Fms-like tyrosine kinase 3 (Flt3) is a receptor tyrosine kinase that plays a crucial role in normal haematopoiesis [15]. Stimulation of Flt3 with its ligand activates phosphatidyl-inositol-3 kinase (PI3K)/Akt and Ras-Raf-MEK-ERK signaling, which regulate the proliferation and survival of hematopoietic progenitor cells [21, 33]. Activity-enhancing mutations of Flt3 are key drivers of acute myeloid leukemia (AML) and can be found in 15–35% of AML patients [32]. Several TKIs targeting aberrant Flt3 signaling to decrease relapse rate and improve survival from AML have been developed. Quizartinib (AC220) is a second-generation inhibitor specifically designed to inhibit mutated Flt3 [31, 41]. The drug produced promising results in several clinical trials as single agent for AML treatment and was well tolerated [5, 6, 29]. However, quizartinib also possesses activity against wild-type Flt3, as it is the case for many other Flt3-specific and multi-targeting TKIs that are in use for leukemia and solid tumor therapy [35]. Importantly, cardiac sides effects including dyspnea, cardiomyopathy and heart failure occur under multi-targeting TKIs [3] and have also been reported under quizartinib [10].

Flt3 is expressed in the heart and upregulated after myocardial infarction (MI) in mice [1]. We found that stimulation of Flt3 with recombinant Flt3 ligand exerts cytoprotective effects and improves post-MI remodelling and function in mice when administered at therapeutic concentrations into the infarct border zone [27]. Flt3 ligand also activated Akt-dependent pro-survival signalling and reduced oxidative stress-induced apoptosis in cultured cardiomyocytes [27]. However, knowledge on the cardiovascular toxicity of Flt3-targeting TKIs is scarce and their effects in patients with cardiovascular diseases are largely unknown. We therefore sought to test whether and how pharmacological inhibition of Flt3 affects cardiac remodelling and function in mice undergoing a MI while receiving quizartinib.

## Materials and methods

### Animals and in vivo model

Studies were conducted in 5- to 7-week-old male C57BL/6NRj mice (Janvier). Housing and animal care were carried out at the Animal Facility of the Department of Biomedicine, University Hospital Basel, Switzerland. Mice were housed for at least one week upon arrival before study inclusion. All procedures were performed according to the guidelines of the Swiss Federal Act on Animal Protection and the National Institutes of Health Guide for the Care and Use of Laboratory Animals and approved by the Veterinary Department of Basel, Switzerland (license numbers 26,840 and 32,517).

### Quizartinib dosing and treatment

Quizartinib (Selleck Chemicals, # S1526) was diluted via sonication in 15% of the polyanionic beta-cyclodextrin Captisol® (CyDex pharmaceuticals, # RC-0C7-100) to the concentration of 2 mg/ml and administered through oral gavage once daily at a dosage of 10 mg/kg/day based on preclinical studies [41], in which a maximum plasma level of 3.8 µM was reached within 2 h after drug administration [41]. The drug treatment study in healthy mice included a total of 48 mice, which were randomly assigned to receive quizartinib or vehicle (captisol) for four weeks. The infarction study included 105 mice, which were also randomly assigned to the quizartinib- or vehicle group. In 81 animals treatment was started 21 consecutive days before surgery, whereas in the remaining mice, the pre-treatment phase was shortened to 5 days before surgery after not seeing any differences between quizartinib- and vehicle-treatment in healthy mice. Mice were then again randomly assigned to sham or MI surgery. Eight animals (4 on vehicle and 4 on quizartinib) died immediately before or during surgery. All surviving animals continued drug or vehicle administration for another 7 consecutive days after surgery.

### Echocardiography

Transthoracic echocardiography was conducted after drug or vehicle treatment, before surgery, and at one week post-surgery using a Vevo 2100 Ultrasound device (VisualSonics, Toronto, ON, Canada) equipped with a MS-550 linear-array probe working at a central frequency of 40 MHz. Mice were anesthetized with 5% isoflurane in pure oxygen for induction and 2% isoflurane for the maintenance through a nose cone. The body temperature was maintained at 37 °C as measured by a rectal thermometer on a warming pad. Chest hair was removed with a depilatory cream. Parasternal long-axis views in B-mode and parasternal short-axis views at mid-papillary muscle level in both B- and M-mode were acquired. Data were transferred to an offline computer and analysed with Vevo 2100 1.6.0 software by an investigator, who was blinded for the experimental details including treatment allocation and surgical group. Left ventricular (LV) anterior (LVAW) and posterior (LVPW) wall thickness and internal dimensions (LVID) were derived from the measurement of M-Mode. Values were averages of three cardiac cycles. Left ventricular ejection fraction (EF) was calculated from derived volumes (Vol), which are computed based on the Teichholz formulas: LV Vol;d = (7.0 / (2.4 + LVID;d)) × LVID;d^3^, LV Vol;s = (7.0 / (2.4 + LVID;s)) × LVID;s^3^, EF = 100 × ((LV Vol;d – LV Vol;s) / LV Vol;d), whereby d denotes diastole and s denotes systole. LV mass was calculated based on the corrected cube formula: LV mass = 1.053 × [(LVID;d + LVPW;d + LVAW;d)^3^ – LVID;d^3^] × 0.8. Speckle tracking echocardiography (STE) was used to assess the longitudinal and circumferential myocardial wall deformation (strain). For this purpose, the endocardium was manually traced on the cine loops at parasternal long-axis and short-axis view, respectively. Manual adjustments were made when needed. Endocardial strain values were calculated with the strain package (VevoStrain™, Vevo 2100 version 1.6.0). All parameters were measured in three consecutive cardiac cycles and values were averaged.

### Myocardial infarction (MI) model

Mice treated for 5 days to three weeks with quizartinib or vehicle were randomly assigned to undergo sham or MI surgery. Before surgery, animals were anesthetized with intraperitoneal injection of ketamine/xylazine/acepromazine (65/15/2 mg/kg) in 0.9% saline. Occlusion of the left anterior descending (LAD) coronary artery was conducted according to previous reports [7, 34]. Briefly, anesthetized mice were intubated endotracheally and connected to a rodent MiniVent ventilator (Model 845, Harvard Apparatus, Holliston, MA, USA). A small chest incision was made between the third and fourth left intercostal space, which provided access to the beating heart. After carefully cutting open the pericardium, the heart was exposed and the LAD was permanently occluded with a 8/0 polypropylene suture (B. Braun, Germany). The successful ligation was verified by immediate discoloration of the affected myocardium. Then, the chest was closed, the lungs were reinflated, and a subcutaneous injection of 100 µl 0.9% saline was given for liquid compensation. The mice were then transferred to a warmed chamber for waking-up. Sham-operated mice underwent thoracotomy, pericardial incision and cardiac muscle puncturing by pulling an 8/0 suture through the muscle around the LAD without ligation. After recovery from operation, subcutaneous injections of buprenorphine (0.1 mg/kg in 0.9% NaCl solution) were given every 6 h during daytime for two consecutive days. An additional dose of buprenorphine was provided *per os* during the night (for two consecutive nights) in the drinking water (9 µg/ml). Changes of body weight, physical condition and behaviour were monitored and recorded daily. The mice were sacrificed immediately after the last echocardiography for heart collection.

### Perfusion and paraffin embedding

At the time of sacrifice 350 mM potassium chloride (KCl) diluted in water was injected intravenously through the jugular vein to arrest the heart in diastole. The thoracic cavity was opened with scissors. A small incision was made on the right atrium to release the blood. A 23G needle, which was connected to a syringe attached to a pressure-controlled perfusion pump, was gently inserted through the apex into the LV. Afterwards, the heart was perfused with 15 mL of cold 4% paraformaldehyde (PFA) under a pressure between 70 and 100 mmHg. The heart was cut free from the main vessels and surrounding tissues. The atria and the right ventricle were removed and the weight of the LV was measured. The value was normalized to the tibia length. The right ventricle was discarded and the LV was fixed in formaldehyde (16%) diluted to 4% in PBS at 4 °C overnight. The next morning the hearts were transferred to 70% EtOH for approximately 6–7 h and then cut transversally into 2 sections with a razor blade, processed and embedded in paraffin according to routine procedures. The embedded tissue was cut into 4 μm sections on a Microm HM 340E Electronic Rotary Microtome (ThermoFisher Scientific, Michigan, USA) and used for histology.

### Picrosirius staining

The tissue was incubated at 55 °C for 30 min, then deparaffinized and rehydrated, and treated with haematoxylin solution, Gill No.3 (Richard-Allan Scientific, #GHS316) for 12 min followed by washing with running tap water. Afterwards, it was dipped into 100% EtOH for 1 min and incubated in Sirius Red f3b (diluted in 0.1% picric acid) for 1 h. The tissue was then dehydrated and mounted in embedding medium (J.T.Baker, #E3921). The stained tissue was scanned using brightfield settings on a Widefield Nikon Ti2 microscope and analysed with Fiji applying colour deconvolution. Fibrosis was quantified at the mid-ventricular level as % picrosirius positive tissue.

### Terminal deoxynucleotidyl transferase dUTP nick end labelling (TUNEL) assay on tissue

The tissue was incubated at 55 °C for 30 min, then deparaffinized and rehydrated and permeabilized using 10 mM TrisHCl and 20 µg/ml proteinase K (ThermoFisher Scientific, #EO0491) for 25 min. The tissue was then washed with tris-buffered saline (TBS) and incubated with in situ cell death detection kit, Fluorescein (Roche, # 11,684,795,910), for 1 h at 37 °C in the dark. It also was stained with monoclonal mouse α-sarcomeric-actinin (1:20 Sigma, #A7811) followed by secondary antibody goat anti-mouse Alexa Fluor 568 (1:800, ThermoFisher Scientific, #A11004). The nuclei were stained with 4’,6-diamidino-2-phenylindole (DAPI) (Invitrogen, #D1306). The tissue was mounted with home-made anti-fading medium (0.22 M 1,4-Diazabicyclo[2.2.2]-octane (DABCO) (Sigma-Aldrich, #D27802), 4.3 mM polyvinyl alcohol 4–88, and 133.3 mM Tris-HCl in 33% v/v glycerol/aqueous solution). Apoptotic cells in the infarct border zone (160 μm) were detected by a Widefield Fluorescence Nikon Ti2 microscope (magnification 20×) and quantified with NIS-Elements AR Analysis 5.11.00 64-bit (Nikon).

### Cleaved caspase-3 staining

Deparaffinized and rehydrated tissue was processed for antigen retrieval using Antigen Unmasking Solution (Vectorlabs, #H3300) at 97 °C for 25 min, left to cool down for 1 h, then permeabilized with 0.2% Triton X-100 in phosphate-buffered saline (PBS) for 45 min, washed with PBS and blocked with 0.2% gelatine and 0.5% bovine serum albumin (BSA) in PBS for 30 min. The tissue was incubated with polyclonal rabbit anti-mouse cleaved caspase-3 primary antibody (1:100 Cell Signalling, #9661) overnight at 4 °C, washed with PBS the next day, blocked with 0.2% gelatin and 0.5% BSA for 10 min and then incubated with HRP-anti-rabbit-IgG (Dako, # P0448) for 1 h at room temperature. Then, the tissue was washed with PBS and the 3,3’-diaminobenzidine tetrahydrochloride (DAB) solution (ThermoFischer Scientific, #34,002) was added for 10 min, following washing with tap water, haematoxylin staining and dehydration. Cleaved caspase-3 positive cells were detected using brightfield settings on a Widefield Nikon Ti2 microscope (magnification 20x) and manually quantified with Fiji by counting the brown-stained nuclei.

### Wheat germ agglutinin (WGA) staining

Deparaffinized and rehydrated tissue was processed for antigen retrieval as described, then washed with TBS 0.1% Tween 20 and blocked with 10% goat serum (Life Technologies, #50-062Z) for 1 h. Heart tissue was stained with FITC-conjugated lectin from Triticum vulgaris (wheat germ agglutinin (WGA)) (1:100 Sigma, #4895). Cell nuclei were detected with DAPI (Invitrogen, #D1306). Tissue was mounted with SlowFade Antifade kit (Invitrogen, #S2828). The stained samples were visualized using a Widefield Fluorescence Nikon Ti microscope (magnification 20x) and automatically quantified with NIS-Elements AR Analysis 5.11.00 64-bit (Nikon) applying Bright Spot Detection and thresholding FITC (green) channel.

### Cell culture and treatment

The rat embryonic myoblast cell line H9c2 was purchased from the European Collection of Authenticated Cell Cultures (ECACC, #88,092,904). The cells were cultured in Dulbecco’s Modified Eagle Medium (DMEM) high glucose L-glutamine, pyruvate (Gibco, #31885-023) supplemented with fetal bovine serum (FCS) to a final concentration of 10% (Gibco, #102170-106), 25 mM HEPES (Gibco, #15630-056), and 1% penicillin and streptomycin (Gibco, #15,140) in a humidified incubator containing 5% CO_2_ at 37 °C. Subconfluent cells (80–90%) were trypsinized and plated in 10% FCS-containing medium in 35 or 60 mm dishes or 96-well plates. The medium was changed to serum-free medium the night before treatment with different concentrations of quizartinib and H_2_O_2_ at different time points according to the set-up of each experiment.

### Neonatal rat ventricular myocyte (NRVM) isolation

NRVM were isolated from 1–3 days old Sprague-Dawley rats as previously described [16]. In brief, pups were sacrificed by decapitation and the hearts were quickly removed. Atria, vessels and lung tissue were removed and ventricles were transferred into Trypsin-EDTA 0.05% (Gibco, #25300) and incubated at 4°C on a slow shaker overnight. Hearts were washed with 7 ml of DMEM low glucose L-glutamine, pyruvate (Gibco, #31885-023) supplemented with FCS to a final concentration of 7% (Gibco, #102170-106), 25 mM HEPES (Gibco, #15630-056), 1% penicillin and streptomycin (Gibco, #15140) and then digested with collagenase type 2 (36 mg/50 ml HBSS) (Worthington, #LS004174) via shaking in the water bath at 37°C. The cell suspension was filtered through a 100 µm cell strainer to remove undigested tissue. Cells were resuspended in FCS-containing DMEM and preplated in a cell culture flask for 1 hr at 37°C to remove most of fibroblasts and endothelial cells. Preplating was repeated one more time. Afterwards, cells were counted using the Neubauer chamber and plated in FCS-containing DMEM with 100 µM of 5-bromo-2’-desoxyuridine (BrdU) (Sigma, #B5002) to prevent proliferation of non-myocytes. The cells were kept in FCS-containing DMEM for 24 h and then incubated in serum-free DMEM overnight before use.

### Cell viability assay

Cell viability was measured based on the 3-(4,5-dimethylthiazol-2-yl)-2,5-diphenyltetrazolium bromide (MTT) assay according to the Cell Proliferation Kit I protocol from Roche (#11-465-007-001). H9c2 and NRVM cells were seeded in a 96-well plate at a density of 4 × 10^3^ and 4 × 10^4^ cells/well, respectively. The following day the medium was changed to serum-free and the cells were incubated overnight. After treatment with 200 nM-20 µM quizartinib for up to 72 h depending on the experimental set-up, 10 µl of the MTT labeling reagent (final concentration 0.5 mg/ml) was added to each well and the plate was incubated for 4 h at 37 °C. Then 100 µl of the Solubilization solution was added into each well and the plate was incubated overnight at 37 °C. The next day absorbance was measured using the Synergy H1 Hybrid Multi-Mode Reader (BioTek, Switzerland) at 570 nm.

### Annexin V and propidium iodide (PI) staining

H9c2 cells were treated with 2–20 µΜ quizartinib for 24 h, H_2_O_2_ for 6 h or both. Following treatment, the supernatant (containing detached cells) was harvested and the remaining adherent cells were trypsinized using Trypsin-EDTA 0.05% (Gibco, #25,300) for 1–3 min, followed by addition of 10% FCS-containing DMEM for Trypsin deactivation. The supernatant and the trypsinized cells were pooled and centrifuged at 1500 rpm for 5 min. The pellet was washed with PBS, followed by binding buffer, and resuspended in binding buffer with FITC-Annexin V (2.25 µg/ml) (BioLegend, #640,905) and incubated for 10–15 min in the dark at room temperature. Cells were then washed and resuspended again in binding buffer and PI (5 µg/ml) (Merck, #P-4864) staining mix was added. Cell death was assessed using a BD LSRFortessa™ cell analyzer (BD Biosciences). Analysis of flow cytometry data was performed with FlowJo Software (version 10.8.2, BD Life Sciences).

### Cell signaling

H9c2 cells were treated with 20 µM quizartinib for 2 h followed by 100 µM H_2_O_2_ (30% w/w, H2O2) (Merck # H1009) for 15 min, 30 and 60 min. Cell lysates were collected to quantify phosphorylated and total Akt, ERK and p38 MAPK by Western Blotting.

### Western blot analysis

Protein samples were obtained from cell lysates using RIPA buffer (Merck, #R0278) containing PhosSTOP (Merck, #04-906-845-001) and Complete Protease Inhibitor Cocktail (Merck, #11-697-498-001). Protein samples were reduced with dithiothreitol and heated at 95 °C with shaking. Samples were loaded on 10% SDS-Page and run at 110 V. Protein samples were transferred onto PVDF membranes previously activated with methanol. After blocking for 1 h with 4% Bovine Serum Albumin (BSA) (Merck, #A7906), the membrane was incubated overnight with Phospho-Akt(S473) (cell signaling, #4058), Phospho-p42/44 MAPK (Erk1/2; T202/Y204) (cell signaling, #9101) or Phopsho-p38 MAPK (T180/Y182) (cell signaling, #9211) diluted in 4% BSA. Total Akt (cell signaling, #9272), ERK (cell signaling, #9102) and p38 MAPK (cell signaling, #9212) were used for normalisation. Horseradish peroxidase-conjugated antibodies (Jackson Immuno Research) were used as secondary antibodies with incubation for 1 h. SuperSignal™ West Pico PLUS Chemiluminescence Substrate (Thermo Scientific, #34,580) was used for protein detection. Quantifiation of protein was done using Fusion FX7Edge software. For multiple stainings the membranes were re-blotted after stripping with Re-Blot Plus Strong Solution (Merck, #2504).

### Terminal deoxynucleotidyl transferase dUTP nick end labelling (TUNEL) assay on cells

H9c2 cells were plated in 35 mm dishes at a density of 125’000 cells/dish over uncoated coverslips. After 24 h, the medium was changed to serum-free medium for another 24 h. Cells were then treated with 10 µM SB203580 (p38 MAPK inhibitor, Sigma-Aldrich # S8307) for 1 h, and quizartinib was added at a concentration of 20 µM for another 22 h, followed by exposure to 100 µM H_2_O_2_ for 6 more hrs. Cells were subsequently washed twice with ice-cold PBS, air-dried, fixed with freshly prepared 4% paraformaldehyde in PBS (pH 7.4) for 1 h at room temperature and permeabilised with 0.1% Triton X-100 in 0.1% sodium citrate (Sigma-Aldrich, # C3674) for 3–5 min on ice. The cells were then washed again with PBS and incubated with in situ cell death detection kit, Fluorescein (Roche, # 11,684,795,910) for 1 h at 37 °C in the dark.

### Data presentation and statistical analyses

D’Agostino-Pearson normality test was applied to test for Gaussian distribution for in vivo and ex vivo data and Shapiro Wilk test for in vitro data. Parametric tests were used for normally distributed and non-parametric tests for non-normally distributed data. Unpaired t-test or Mann Whitney-U test were used to compare two groups and ordinary one-way ANOVA or Kruskal-Wallis followed by Sidak’s and Dunn’s, respectively, to compare multiple groups. Data from in vivo mouse experiments are presented as violin blots in the Figures and mean ± SEM in the table. For echocardiography data, two-way ANOVA followed by Tukey’s (comparison between groups) or Sidak’s (comparison between time points) multiple comparison tests were used. Data from in vitro studies are presented as mean ± SEM and significance was tested by ordinary one-way ANOVA with Sidak’s multiple comparison test for normally distributed data and Friedman multiple comparison test for non-normally distributed data. Kaplan Meier survival curves were compared using Gehan-Breslow-Wilcoxon test. Statistical analyses were performed with GraphPad Prism version 8 software (GraphPad).

## Results

### Quizartinib does not lead to changes in cardiac morphology or function after four weeks of therapy in healthy mice

Forty-eight mice were randomly assigned to receiving quizartinib (10 mg/kg/day) or vehicle (15% captisol) by daily gavage for four weeks and echocardiography was performed at the end of the four-week treatment period. After four weeks of therapy, mice on quizartinib did not show any differences in cardiac dimensions or functional parameters as compared to vehicle-treated mice (Supplemental Table 1), suggesting that in healthy mice, quizartinib per se does not affect cardiac morphology or function.

### Quizartinib does not worsen survival or cardiac function at one week after Myocardial Infarction

**Fig. 1:**
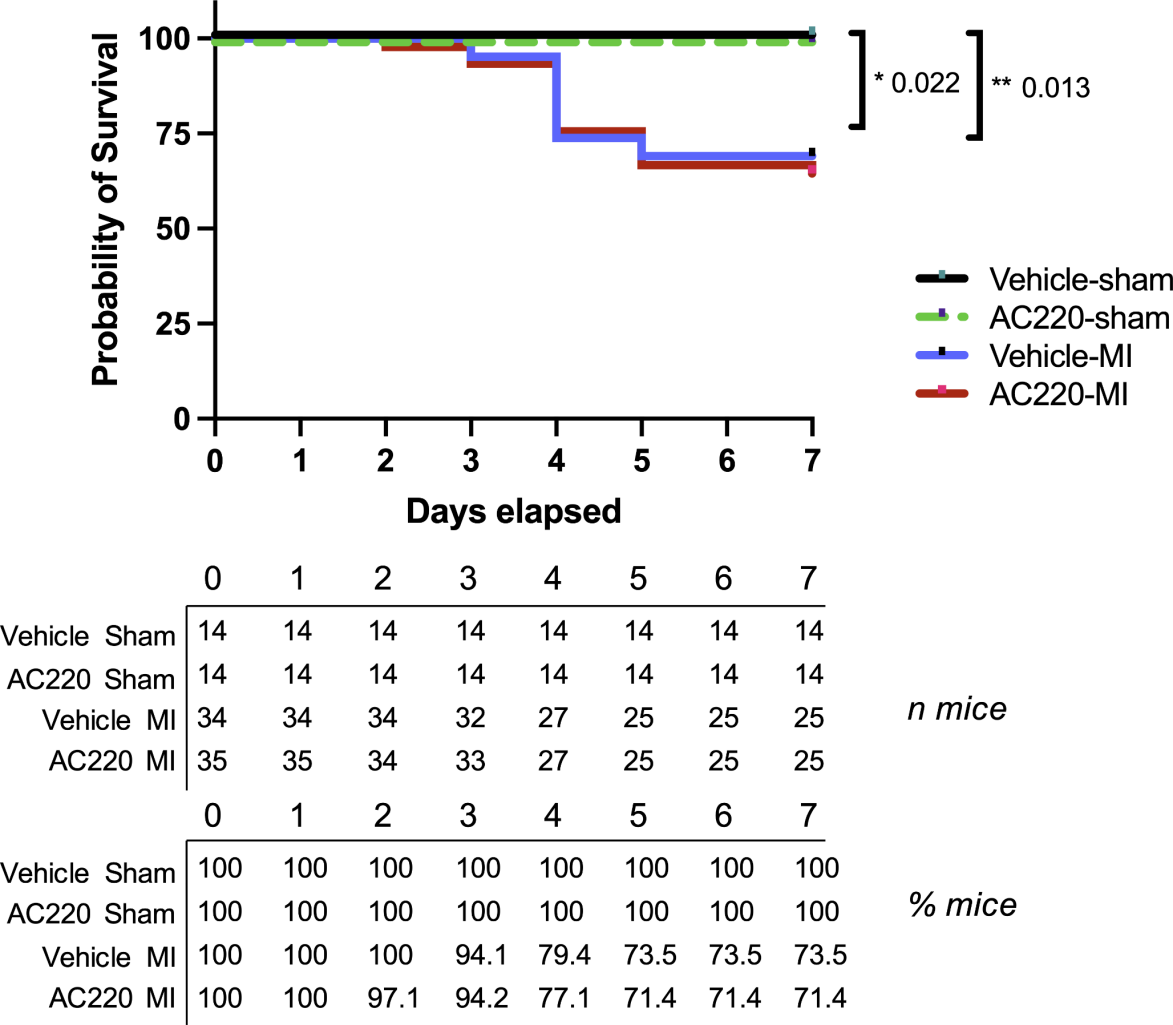
Post-procedural survival up to 7 days. Kaplan Meier survival curves for C57BL/6NRj mice treated with quizartinib (AC220) or vehicle and undergoing sham surgery or MI up to 7 days. Excluded are the eight mice (four on quizartinib and four on vehicle), which died before or during surgery. Data are represented as survival of animals per time point. Vehicle-Sham n = 14, AC220-Sham n = 14, Vehicle-MI n = 34; AC220-MI n = 35. Groups were compared using Gehan-Breslow-Wilcoxon test; *p = 0.022 Vehicle-Sham vs. Vehicle-MI; **p = 0.013 AC220-Sham vs. AC220-MI. AC220: quizartinib

Given the cardioprotective effects of Flt3 stimulation in the infarcted heart [27], we next sought to test whether pharmacological inhibition of Flt3 during MI leads to more pronounced cardiac injury and dysfunction and worse outcome. Five- to seven-week-old mice were pretreated for five days or three weeks with either quizartinib or vehicle, then randomly assigned to LAD ligation or sham surgery, and quizartinib or vehicle treatment was continued for another week. Echocardiography was performed before and at one week after surgery (Supplemental Figure S1). Less animals survived after MI than after sham surgery, irrespective of quizartinib treatment, but mortality was not significantly different between the treatment groups (Fig. 1).

**Fig. 2:**
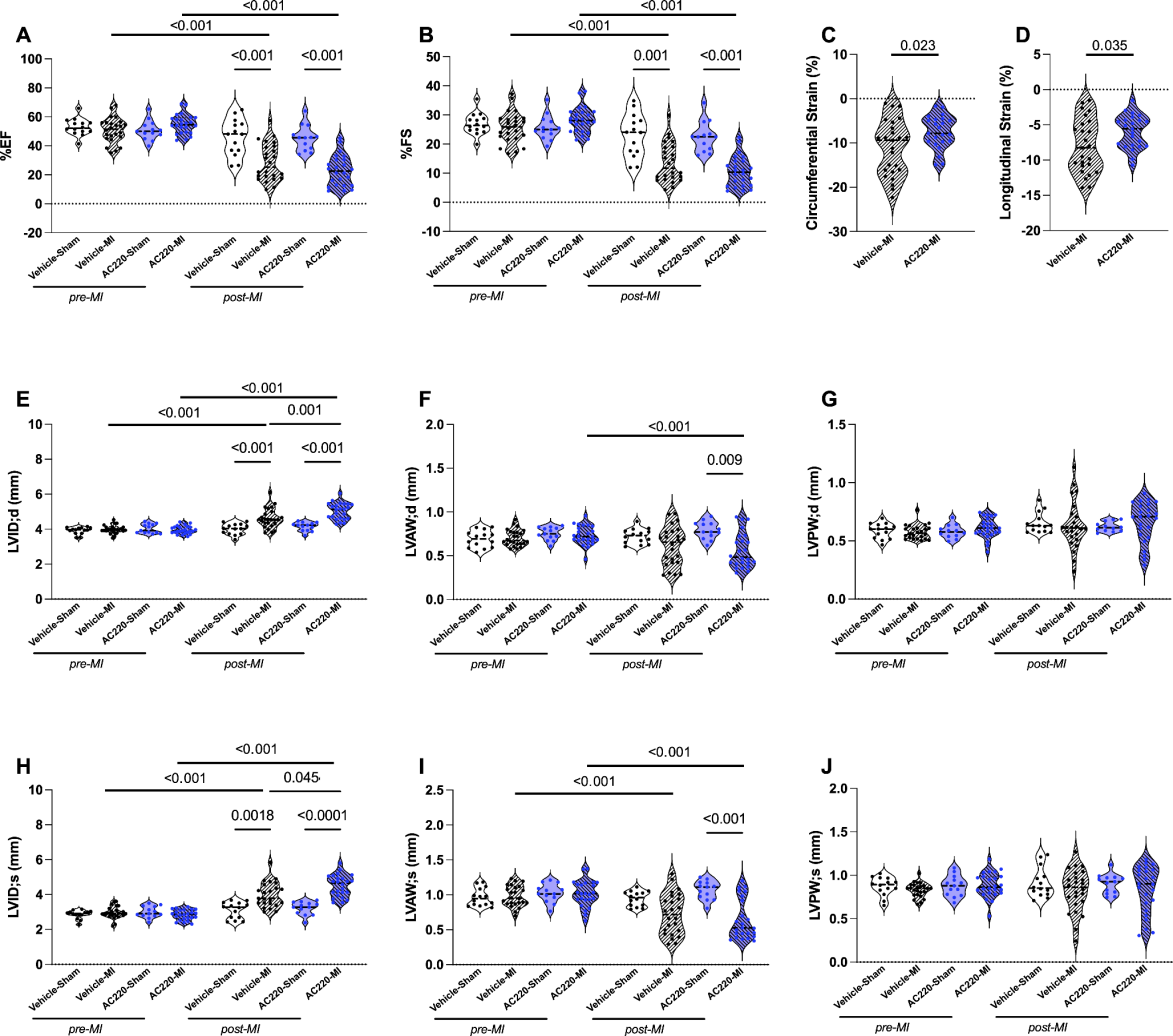
Cardiac function and morphology by echocardiography at baseline and at one week post-MI. Serial echocardiography was performed before and one week after surgery. Groups were compared using 2-way ANOVA followed by Tukey’s and Sidak’s multiple comparison test for comparisons between groups and between time-points for the same group, respectively. **(A)** Cardiac function in terms of left ventricular ejection fraction (EF). **(B)** Cardiac function in terms of fractional shortening (FS). (**C** and **D**) Cardiac function in terms of circumferential and longitudinal strain. (**E** and **H**) Left ventricular inner diameter at end-diastole (LVID;d) and end-systole (LVID;s). (**F** and **I**) Wall thickness of the left anterior wall at end-diastole (LVAW;d) and end-systole (LVAW;s). (**G** and **J**) Left ventricular posterior wall at end-diastole (LVPW;d) and end-systole (LVPW;s). Vehicle-Sham n = 14, AC220-Sham n = 14, Vehicle-MI n = 25, AC220-MI n = 25. AC220: quizartinib

One week after MI, global systolic function as assessed by left ventricular ejection fraction (LVEF) and fractional shortening (FS) was significantly decreased in both quizartinib- and vehicle-treated mice compared to before surgery. Likewise, LVEF and FS were significantly lower after MI compared to sham-operated mice for both treatment groups, but there was no difference in LVEF or FS between vehicle-treated and quizartinib-treated infarcted mice (Fig. 2A and B). To further assess cardiac function after MI with more sensitivity, myocardial strain was measured by STE. Both circumferential and longitudinal strain were significantly lower in quizartinib-treated compared to vehicle-treated infarcted hearts (Fig. 2C and D).

### Quizartinib aggravates maladaptive remodeling post-MI

The weakening of the infarcted wall may lead to cardiac dilation. Accordingly, one week after MI, the LV internal diameters (LVID) were increased in quizartinib- and vehicle-treated mice compared to before surgery and compared to sham-operated mice. In addition, LVID both at end-diastole and at end-systole were significantly larger in the quizartinib- than in the vehicle-treated infarcted mice (Fig. 2E and H). MI leads to thinning of the infarcted myocardial wall. Left anterior wall thicknesses (LVAW;d) were decreased in both quizartinib- and vehicle-treated animals at one week post-MI, and this decrease reached statistical significance only for the quizartinib group when compared to both the same mice pre-MI and to quizartinib sham-operated mice. However, there was no significant difference between the two groups (Fig. 2F and I). There were no differences in posterior wall thicknesses between any of the groups, neither for quizartinib treatment nor after MI (Fig. 2G and J). Organs were retrieved from a subset of mice, in which the hearts were not perfusion-fixed. The LV weight to tibia length ratio was non-significantly higher in mice one week after MI compared to after sham surgery, but comparable between the two treatment groups for both sham-operated and MI mice (Supplemental Figure S2).

### Quizartinib enhances apoptosis in the infarct border zone and in the remote myocardium

**Fig. 3:**
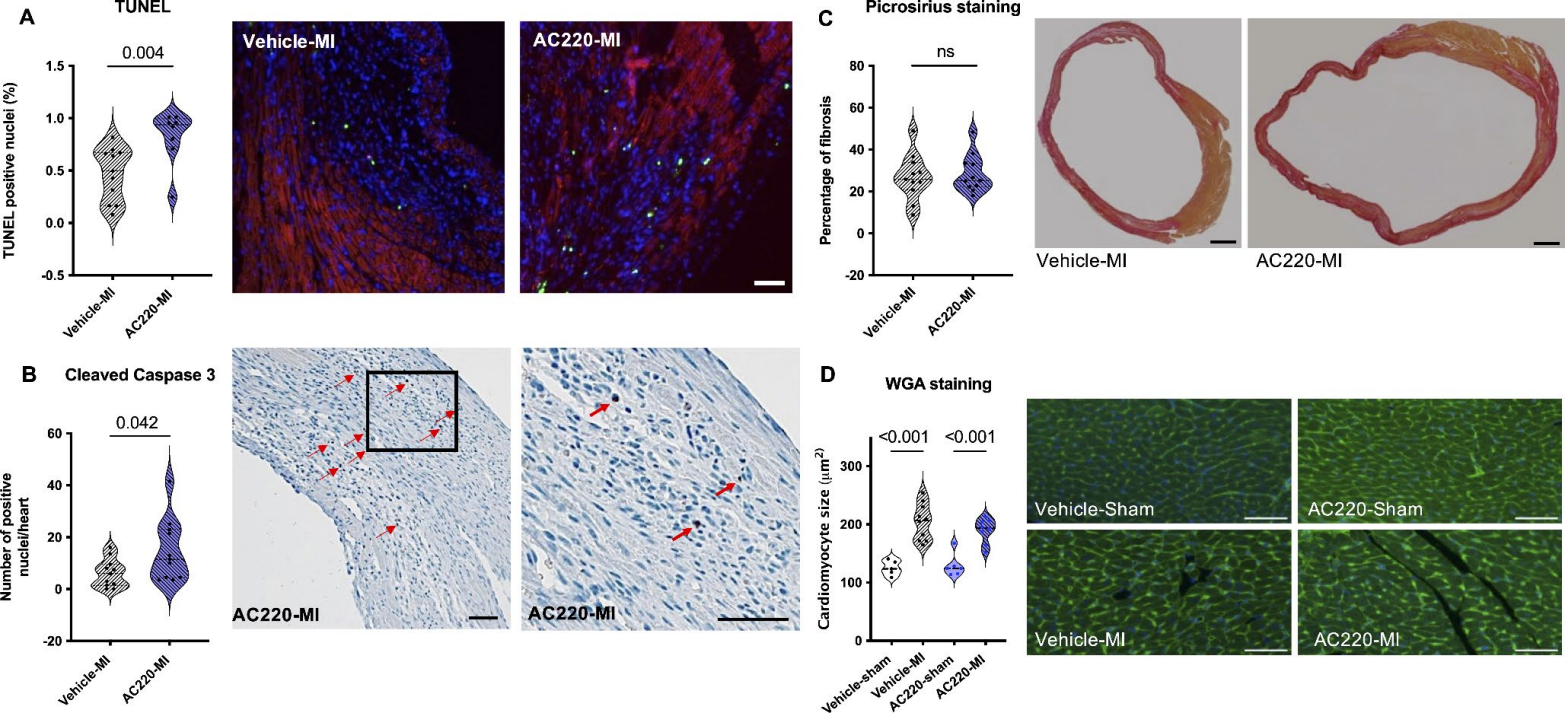
Apoptotic cell death, fibrosis and cardiomyocyte size at one week post-MI. **(A)** TUNEL-positive nuclei in the infarct border zone: quantification and representative microscopic images; red: alpha-sarcomeric actinin, blue: DAPI-positive nuclei; green: TUNEL-positive nuclei; scale bar = 100 μm; nonparametric Mann-Whitney test. **(B)** Cleaved caspase 3-positive cells in the remote myocardium: quantification and representative microscopic image of AC220-MI sample in two magnifications; scale bar = 50 μm; unpaired two-tailed t test. **(C)** Fibrotic area at mid-ventricular level by picrosirius staining: quantification and representative microscopic images of vehicle-MI and AC220-MI; scale bar = 500 μm; unpaired two-tailed t test. **(D)** Cardiomyocyte size assessed by wheat germ agglutinin (WGA) staining: quantification and representative microscopic images; green: cardiomyocyte borders (WGA), blue: nuclei (DAPI); scale bar = 20 μm; unpaired Kruskal-Wallis test. AC220: quizartinib

Infarct damage may be augmented by ongoing apoptotic cell death, which occurs predominantly in the infarct border zone during the first week post-MI [25]. Apoptosis was assessed in the infarct border zone by TUNEL staining and in whole myocardial cross-sections by cleaved caspase 3 staining. Apoptosis was significantly increased in the infarct border zone of quizartinib-treated compared to vehicle-treated mice (Fig. 3A). Moreover, there was also more apoptosis in the remote myocardium as assessed by cleaved caspase 3-positive nuclei, which were significantly more frequent in quizartinib-treated than in vehicle-treated infarcted hearts in the whole cross-section at mid-papillary level (Fig. 3B).

### Quizartinib does not alter the extension of fibrosis at the mid-ventricular level or cardiomyocyte size

To further test whether the enhanced cell death leads to a measurable increase in fibrosis, the deposition of collagen was assessed by picrosirius staining and the percentage of total myocardial tissue that was picrosirius positive was determined at the mid-ventricular level. We observed no difference in the size of the fibrotic area between quizartinib- and vehicle-treated mice at one week after MI **(**Fig. 3C).

We further sought to assess whether there was a difference in compensatory cardiomyocyte hypertrophy by measuring cell size using wheat germ agglutinin (WGA) staining of cellular membranes. Cardiomyocyte size was significantly increased in the hearts of animals that underwent MI surgery compared to sham animals, and this was true for both quizartinib-treated and vehicle-treated mice with no significant difference between the treatment groups (Fig. 3D). These experiments indicate that quizartinib does not appear to enhance fibrosis or compensatory cardiomyocyte hypertrophy after MI.

### Quizartinib decreases cardiomyocyte viability in a dose-dependent manner in vitro

Previous data from our laboratory have shown that activation of Flt3 induces pro-survival signaling and decreases cardiomyocyte apoptosis in vitro [27]. We therefore first examined the effect of quizartinib on primary neonatal rat cardiomyocytes (NRVM) and H9c2 cells. NRVM were cultured for 24 h in 96-well plates containing 7% FCS DMEM medium, then kept in serum-free DMEM medium overnight before being exposed to 20 nM-20 µM quizartinib or DMSO for another 24 h (Supplemental Figure S3A and C). 100 and 500 µM H_2_O_2_ were used as positive control. After 24 h the MTT assay showed that – compared to DMSO – the high concentration range of quizartinib, i.e. 2 µM and 20 µM impaired cardiomyocyte viability in a dose-dependent manner, whereas lower concentrations (20 nM and 200 nM) did not have any effect. In contrast, in H9c2 cells, only the highest concentration of quizartinib decreased viability, whereas all lower concentrations including 2 µM had no effect (Supplemental Figure S3B). These observations suggest that primary cardiomyocytes may be more susceptible to potential toxic effects of quizartinib than the H9c2 cell line. Next, we wanted to explore whether a longer exposure of cells to low concentrations of quizartinib may also decrease cell viability. H9c2 cells were treated with 2 nM-200 nM of quizartinib and cell viability was assessed at 24, 48 and 72 h (Supplemental Figure S2D and E). 1 mM H_2_O_2_ was used as positive and 0.1% FCS as negative control. Cell viability was not affected in response to various low concentrations of quizartinib at any time point. Taken together, these experiments show that high concentrations of quizartinib can reduce cell viability, whereas lower concentrations have no significant effect even over time. Given the comparable responses of NRVM and H9c2 cells to 20 µM quizartinib and in consideration of the three Rs principle of animal experimentation, H9c2 were used for further in vitro studies.

### Quizartinib induces apoptosis in a dose-dependent manner in vitro

**Fig. 4:**
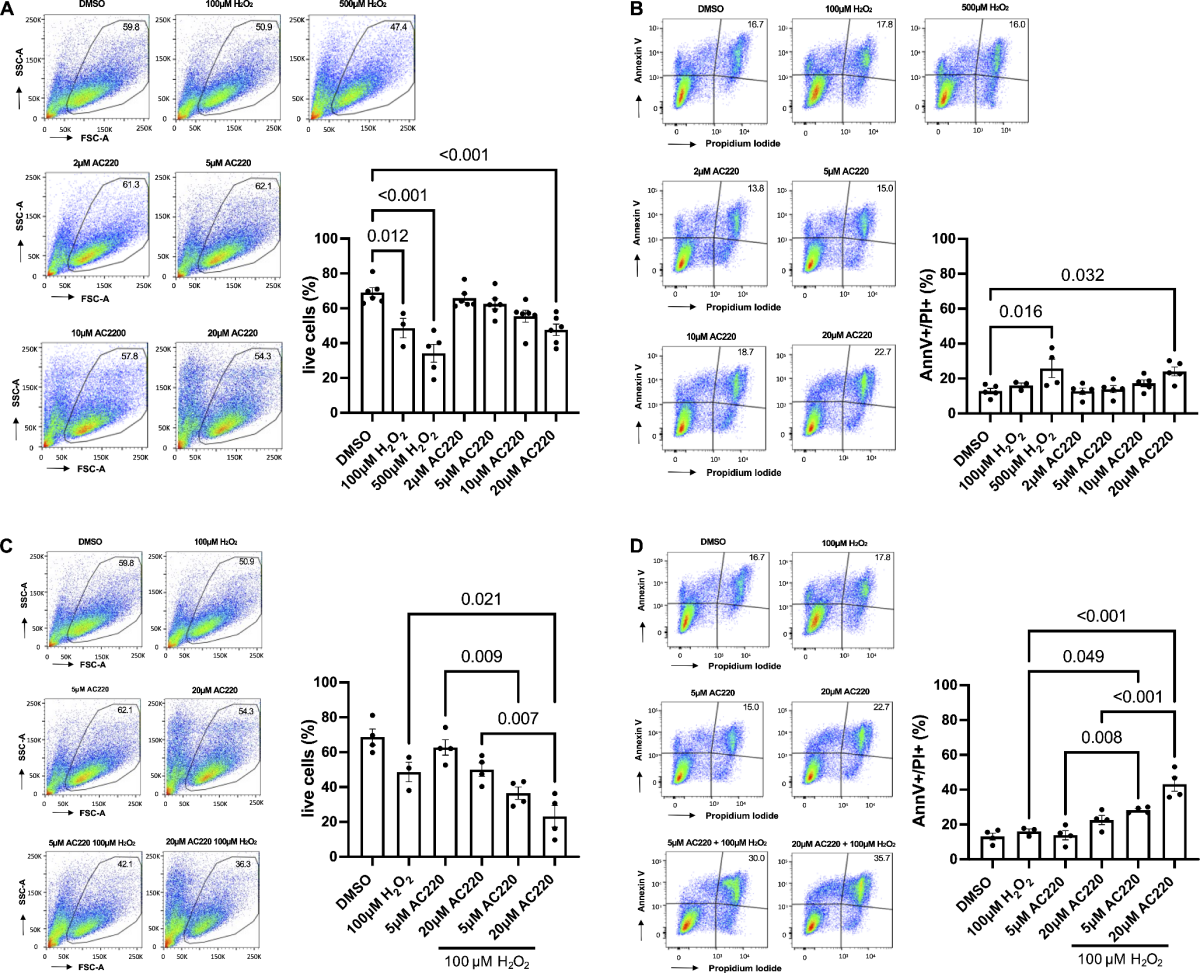
Cell viability and apoptosis under quizartinib alone and together with H_2_O_2_. *Viable and apoptotic H9c2 cells after treatment with different concentrations of quizartinib* in vitro (**A** and **B**). H9c2 cells were treated with quizartinib (AC220) in the dose range of 2 µΜ to 20 µΜ for 24 h. DMSO, and 100 µΜ or 500 µΜ H_2_O_2_ were used as control. Cells were stained with Annexin V and PI staining followed by flow cytometry analysis. The frequencies of viable cells (based on FSC-SSC gating) and of Annexin V^+^PI^+^ cells were assessed. **(A)** Representative flow cytometry plots (left panel) and frequency of viable cells (based on FSC-SSC gating) (right panel). **(B)** Representative flow cytometry plots (left panel), and frequency of Annexin V^+^PI^+^ cells (right panel). The data are presented as mean ± SEM, n = 4–5 independent experiments; one-way ANOVA followed by Sidak’s test. AC220: quizartinib, AnnV: Annexin V, PI: propidium iodide. *Viable and apoptotic H9c2 cells after treatment with different concentrations of quizartinib in combination with H*_*2*_*O*_*2*_ in vitro. H9c2 cells were treated with 5 and 20 µΜ quizartinib (AC220) for 18 h and H_2_O_2_ was added for additional 6 h. DMSO and 100 µΜ H_2_O_2_ were used as control. The frequencies of viable cells (based on FSC-SSC gating) and Annexin V^+^PI^+^ cells were assessed. **(C)** Representative flow cytometry plots (left panel) and frequency of viable cells (based on FSC-SSC gating) (right panel). **(D)** Representative flow cytometry plots (left panel), and frequency of Annexin V^+^PI^+^ cells. The data are presented as mean ± SEM, n = 3–4 independent experiments; one-way ANOVA followed by Sidak’s test. AC220: quizartinib, AnnV: Annexin V, PI: propidium iodide

Quizartinib inhibits cell proliferation and induces apoptosis in leukemia cell lines that are dependent on Flt3 [17]. Because of the decreased cell viability above concentrations of 2 µM in vitro, we examined whether the decrease in cell viability could be attributed to apoptosis. Apoptotic cell death in response to 2–20 µM quizartinib for 24 h was measured in H9c2 cells by flow cytometry using Annexin V and PI double staining. Consistent with the data obtained by MTT, treatment with quizartinib led to a dose-dependent reduction in the frequency of viable cells as assessed by FSC-SSC gating (Fig. 4A). Quizartinib treatment also increased the number of Annexin V^+^PI^+^ cells in a dose-dependent manner, and with a roughly two-fold increase in response to 20 µM compared to DMSO (Fig. 4B). These findings suggest that at least part of the quizartinib-induced loss of cell viability is mediated by apoptosis.

### Quizartinib potentiates H_2_O_2_-induced cell death and apoptosis in vitro

Ischemia/reperfusion injury contributes to apoptotic cell death in the infarct border zone [42], which is mediated by oxidative stress [13]. We therefore pretreated H9c2 cells with 5 and 20 µM quizartinib for 18 h before adding 100 µM H_2_O_2_ for additional 6 h. The combination of quizartinib with H_2_O_2_ markedly enhanced the decrease in viable cells and the increase in Annexin V^+^PI^+^ cells. In fact, H_2_O_2_ on top of 20 µΜ quizartinib approximately led to a doubling of the amount of Annexin V^+^PI^+^ cells versus quizartinib alone and a tripling versus H_2_O_2_ alone (Fig. 4C and D). These findings suggest that quizartinib potentiates oxidative stress-induced apoptosis.

### Apoptosis under quizartinib together with H_2_O_2_ results from potentiation of p38-signaling

**Fig. 5:**
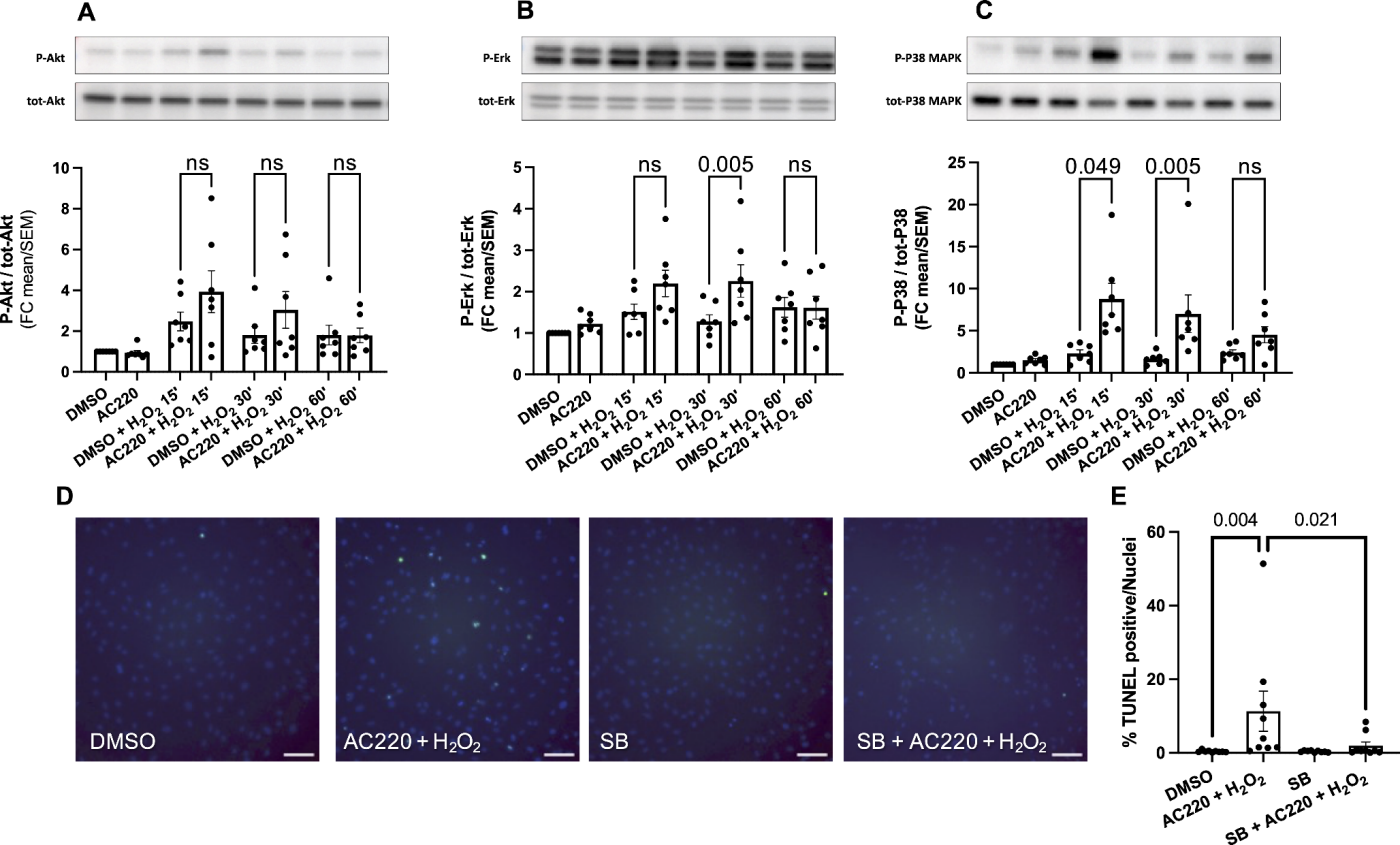
Time-dependent phosphorylation of Akt, ERK and p38 in H9c2 cells after quizartinib treatment and in combination with H_2_O_2_ and role of p38 in apoptosis. H9c2 cells were treated with 20µM quizartinib (AC220) for 2 h and 100 µM H_2_O_2_ was added for additional 15, 30 and 60 min. **(A-C)** Representative images and protein quantification by Western blot analysis of the phosphorylation of Akt, ERK and p38 MAPK. Data are presented as mean ± SEM, n = 7 independent experiments; one-way ANOVA followed by Sidak’s test or Friedman followed by Dunn’s multiple comparisons correction test for normality-failed datasets. **(D-E)** TUNEL-positive nuclei in H9c2 cells: quantification and representative microscopic images; blue: DAPI-positive nuclei; green: TUNEL-positive nuclei; scale bar = 100 μm; Data are presented as mean ± SEM, n = 8 independent experiments; Friedman followed by Dunn’s multiple comparison correction test. AC220: quizartinib; SB: SB203580.

Activation of Akt and the MAPK ERK occur downstream of Flt3 [18]. Both, Akt and ERK, exert cytoprotective effects in various cell types including cardiomyocytes, whereas the MAPK p38 is a known mediator of cardiomyocyte apoptosis in response to ischemia/reperfusion [22]. We therefore sought to test whether the enhanced apoptosis might be due to reduced cytoprotective Akt- and/or ERK-signaling or due to enhanced pro-apoptotic signaling through p38. H9c2 cells were pretreated with 20 µM quizartinib for 2 h, followed by 100 µM H_2_O_2_ for 15, 30 and 60 min. H_2_O_2_ on top of quizartinib, but neither compound alone, significantly induced phosphorylation of p38, which peaked at 15 min. In contrast, only slight and inconsistent increase of Akt- and ERK-phosphorylation could be observed (Fig. 5A-C and Supplemental Figure S4). To further test whether apoptosis under double injury with H_2_O_2_ on top of quizartinib is mediated by p38, cells were pretreated with the p38 inhibitor SB203580 for 1 h before adding 20 µM quizartinib for 22 h and 100 µM H_2_O_2_ for another 6 h. Pretreatment with SB203580 almost completely abolished apoptosis in response to H_2_O_2_ on top of quizartinib (Fig. 5D and E).

## Discussion

Despite the success of small molecule TKIs in the treatment of malignancies, there is an increasing number of studies reporting adverse cardiac events in patients under TKI therapy with systolic dysfunction and heart failure being among the most severe side effects [3, 12, 23, 24, 30]. As in other organs, cardiac homeostasis and function are maintained by a highly coordinated network of molecular signaling pathways. Many of these pathways are common to various different cells and organs. In particular, kinases involved in the regulation of cell survival and proliferation may be part of a shared kinome between cancer cells and the heart [20]. However, our understanding of the specific roles of many cancer drug targets in the healthy and injured heart is limited and side effects of TKIs may occur apparently unforeseeably, when the drug is already in clinical use [38].

In our study we examined the second-generation Flt3 inhibitor quizartinib, which has shown a high potency in the treatment of AML either as a single agent or in combination with conventional chemotherapy regimens [43], but also potentially relevant cardiotoxicity in clinical trials [10]. Because we previously found that Flt3 exerts protective effects in the infarcted heart through the inhibition of apoptotic cell death [27], we focused our studies on cardiomyocyte viability. We found that quizartinib-treated mice had significantly enhanced apoptotic cell death at one week post-MI, and this was the case both in the infarct border zone as well as in the remote myocardium. Apoptotic cell death plays an important role in the progression of maladaptive LV remodeling and dysfunction after MI [8, 11, 25, 28, 42]. Consistent with this notion, quizartinib-treated mice showed larger LV end-systolic and end-diastolic diameters after MI than vehicle-treated mice, suggestive of accelerated maladaptive remodeling. Even if we could not observe significant differences in LVEF and FS between quizartinib- and vehicle-treated mice, this increased apoptosis has the potential to translate into more severe functional impairment at a later stage of chronic ischemic heart disease or upon additional stress to the heart. Earlier studies in mice have shown that although rapidly declining within the first two weeks after MI, cardiac function continues to deteriorate up to at least nine weeks [14], with another group showing slow but progressive worsening of cardiac function even up to 4 months [39]. Although further studies are needed to examine whether quizartinib worsens post-MI cardiac function in the chronic phase of the disease, this assumption is supported by previous observations that apoptotic cell death, even at a very low level, is in itself sufficient to cause cardiomyopathy and heart failure [36] and by the significantly lower myocardial deformation parameters (strain) in quizartinib-treated compared to vehicle-treated mice.

In a previous study by Duran et al. using the multi-targeting TKI sorafenib in mice undergoing MI, sorafenib, which also inhibits Flt3, led to a significant increase in cardiomyocyte death, at least in vitro [9]. Importantly, no differences in cardiac function or infarct size up to two weeks post-MI were observed in mice treated with sorafenib compared to vehicle while undergoing MI [9], which is consistent with our findings. However, there are also important differences between this study and ours. Firstly, whereas they observed a higher mortality of sorafenib-treated mice after infarction, survival of quizartinib- and vehicle-treated mice was comparable in our study. Secondly, sorafenib-induced cardiomyocyte death was mostly attributable to necrosis, and not to apoptosis. We observed an increase in TUNEL-positive and cleaved caspase-3 positive cells in the hearts of quizartinib-treated compared to vehicle-treated mice at one week post-MI. To further differentiate the type of cell death and confirm quizartinib-dependent myocyte apoptosis, in vitro studies were performed on NRVM and H9c2 cells. Whereas these studies showed that a considerable amount of cell death is most likely non-apoptotic, we also found a dose-dependent increase in apoptosis as assessed by Annexin V/PI double-positivity in response to quizartinib.

Consistent with our previous findings that Flt3-activation inhibits hypoxia- and oxidative-stress induced cardiomyocyte apoptosis in vitro and in vivo [27], we observed a potentiation of both apoptotic and non-apoptotic cell death in cardiomyocytes exposed to oxidative stress (H_2_O_2_) in the presence of quizartinib. In addition, the combination of quizartinib and H_2_O_2_ led to a significant increase in pro-apoptotic signaling through p38, whereas neither quizartinib nor H_2_O_2_ alone meaningfully increased p38-phosphorylation, and apoptosis under double injury was p38-dependent. From a translational aspect, these observations are consistent with the multi-hit hypothesis of cardiotoxicity [19], which postulates that additional cardiac stressors are needed to unmask latent cardiotoxicity of cancer drugs. Interestingly, quizartinib is more specific for Flt3 than sorafenib. In fact, sorafenib has the potential to inhibit p38 as an off-target effect [41], which could also explain the absence of apoptosis in the study by Duran et al. [9].

## Study limitations

We followed the mice for one week post-MI. However, longer follow-up might be needed to detect functional differences in terms of LVEF and FS caused by the early enhanced cell death and augmented maladaptive remodeling in quizartinib-treated mice. In addition, LVEF was calculated based on the Teichholz formula, which insufficiently accounts for functional deficits caused by a localized injury such as MI. This may have resulted in lesser sensitivity to detect differences. Because Flt3-signaling is potently anti-apoptotic in leukemia cells, we focused our study on apoptosis. However, other types of cell death, including necrosis and autophagy, likewise play a role in post-MI remodeling. We did not assess signaling in the mouse heart and can therefore not comment on the dynamics of p38 phosphorylation and how it relates to apoptosis in vivo. Although more specific for Flt3 than previous TKIs, quizartinib also targets other receptor TKs including colony stimulating factor 1 receptor [41], stem cell factor receptor (SCFR/c-kit) [17, 41] and platelet derived growth factor receptor (PDGFR) [17, 41]. At least for some of these receptors, cardioprotective effects have been shown [4, 37]. In addition, off-target effects through inhibition of unrelated kinases or unspecific drug toxicity could also contribute to quizartinib-toxicity. Therefore, further studies are needed to delineate whether and how the observed toxicity relates to Flt3-inhibition as opposed to other mechanisms. Finally, the study has been conducted in mice and due to differences in physiology, extrapolations to humans must be done with caution.

## Conclusion

**Fig. 6:**
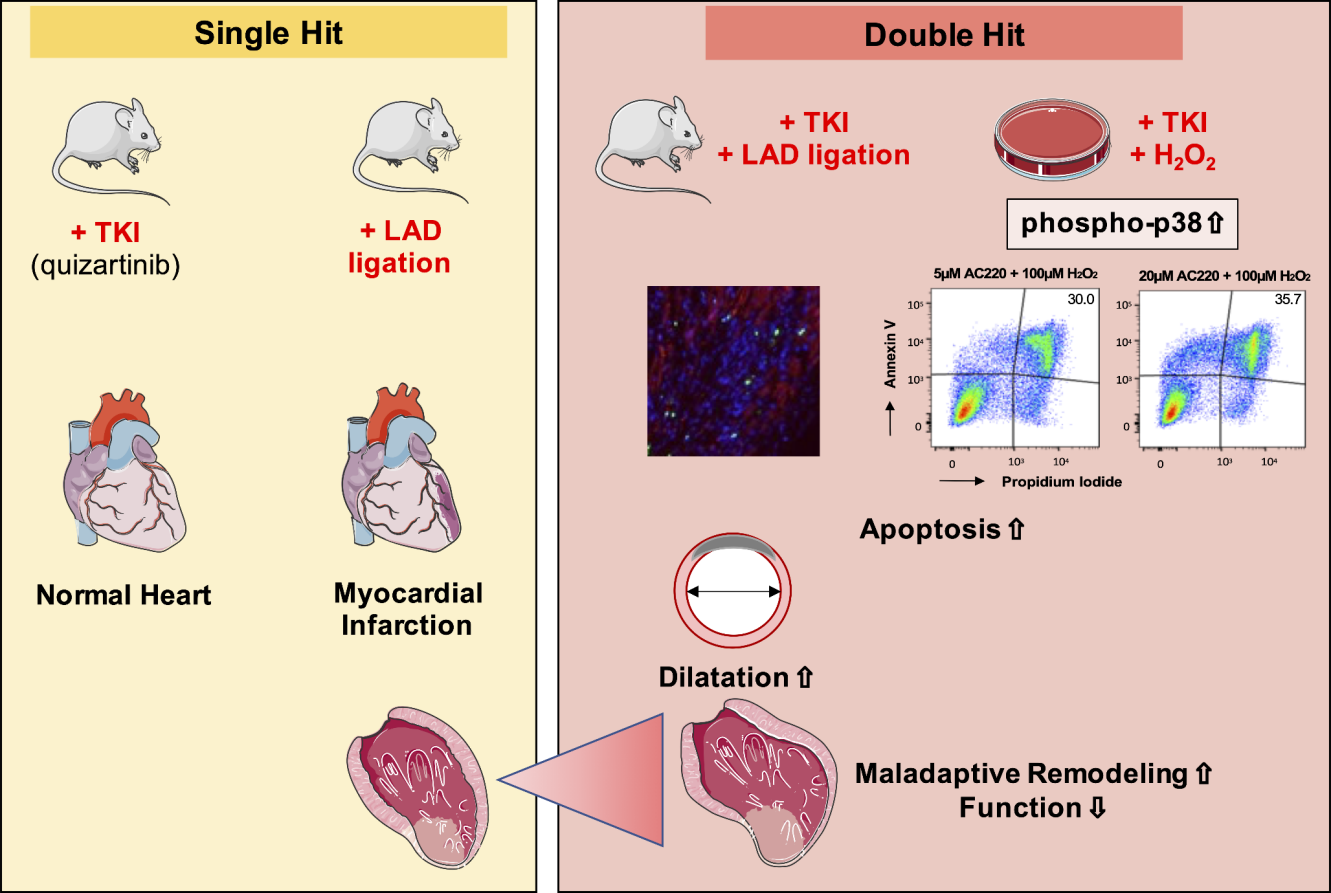
Proposed model for potentiation of maladaptive remodelling under double-injury of myocardial infarction and TKI therapy. For drawing icons from Servier Medical Art, licensed under Creative Commons Attr. 3.0 unported license were used. https://creativecommons.org/licenses/by/3.0/

Quizartinib augments maladaptive remodeling after MI in mice and potentiates oxidative stress-associated apoptotic cell death in vitro and in vivo (Fig. 6), which could explain at least in part some of the toxicity observed in early clinical trials. More work will be needed to understand to what degree these effects are specific for the inhibition of Flt3-signaling or attributable to Flt3-unrelated mechanisms. Better understanding of the mechanisms underlying TKI toxicity is a prerequisite to advance future drug design and to develop cardioprotective strategies to improve the cardiovascular health of cancer patients requiring TKI therapy. Until then, close monitoring of patients with underlying ischemic heart disease undergoing Flt3- or multi-targeting TKI treatment may be warranted.

### Electronic supplementary material

Below is the link to the electronic supplementary material.

Supplementary Material 1

## Data Availability

All data generated or analysed during this study are included in this published article and its supplementary information files.

## Abbreviations

AW: Anterior wall
AML: Acute myeloid leukemia
AC220: Quizartinib
EF: Ejection fraction
Flt3: Fms-like tyrosine kinase 3
FS: Fractional shortening
ID: Inner diameter
LV: Left ventricle, left ventricular
MI: Myocardial infarction
PW: Posterior wall
STE: Speckle tracking echocardiography
TKI: Tyrosine kinase inhibitor
