# Supplementary material for: The Flt3-inhibitor quizartinib augments apoptosis and promotes maladaptive remodeling after myocardial infarction in mice
Source: Apoptosis. 2023 Nov 9;29(3-4):357–71. doi: 10.1007/s10495-023-01911-8 (PMC10873224; doi:10.1007/s10495-023-01911-8)
Supplement: Supplementary file 1 — Supplementary Material 1 [file 10495_2023_1911_MOESM1_ESM.pdf]

Supplemental Table and Figures

The Fms-like tyrosine kinase 3-inhibitor quizartinib augments apoptosis and promotes maladaptive remodeling after myocardial infarction in mice

Daria Monogiou Belik[a], PhD; Riccardo Bernasconi[a], MSc; Lifen Xu[a], PhD; Giacomo Della Verde[a], PhD; Vera Lorenz[a], MSc; Vivienne Grüterich, MSc; Melania Balzarolo[a], PhD; Michika Mochizuki[a], PhD; Otmar Pfister[a,b] , MD, and Gabriela M. Kuster[a,b], MD

[a] Myocardial Research, Department of Biomedicine, University Hospital Basel and University of Basel, Basel, Switzerland; [b] Department of Cardiology, University Hospital Basel, Basel, Switzerland

Brief Title: Quizartinib aggravates post-infarct remodeling

***Correspondence:** Gabriela M. Kuster, M.D., Myocardial Research, Department of Biomedicine, University of Basel and University Hospital Basel, Hebelstrasse 20, 4031 Basel, Switzerland, Tel: ++41 61 328 77 36, Fax: ++41 61 265 45 98, Email: Gabriela.Kuster@usb.ch

Supplemental Table

***Supplemental Table 1**. Echocardiographic parameters after four weeks of quizartinib- or vehicle treatment of healthy mice.*

	**Vehicle**	**AC220**
**N**	24	24
**Body Weight (g)**	26.2 ± 0.2	25.8 ± 0.2
**HR (BMP)**	491 ± 11	481 ± 13
**EF (%)**	56.688 ± 1.876	56.766 ± 1.582
**FS (%)**	29.542 ± 1.246	29.516 ± 1.028
**LVAW; d (mm)**	0.828 ± 0.013	0.851 ± 0.015
**LVID; d (mm)**	3.899 ± 0.045	3.945 ± 0.032
**LVPW; d (mm)**	0.616 ± 0.012	0.628 ± 0.013
**LVAW; s (mm)**	1.192 ± 0.025	1.187 ± 0.028
**LVID; s (mm)**	2.755 ± 0.073	2.785 ± 0.056
**LVPW; s (mm)**	0.971 ± 0.027	1.005 ± 0.026

Data are presented as Mean ± SEM, none of the comparisons was statistically significant (unpaired student's t-test). AC220: quizartinib, HR: heart rate, BMP: beats per minute, EF: ejection fraction, FS: fractional shortening, LVAW: left ventricular (LV) anterior wall, LVID: LV inner diameter, LVPW: LV posterior wall, d: diastole, s: systole.

Supplemental Figures

Supplemental Figure S1

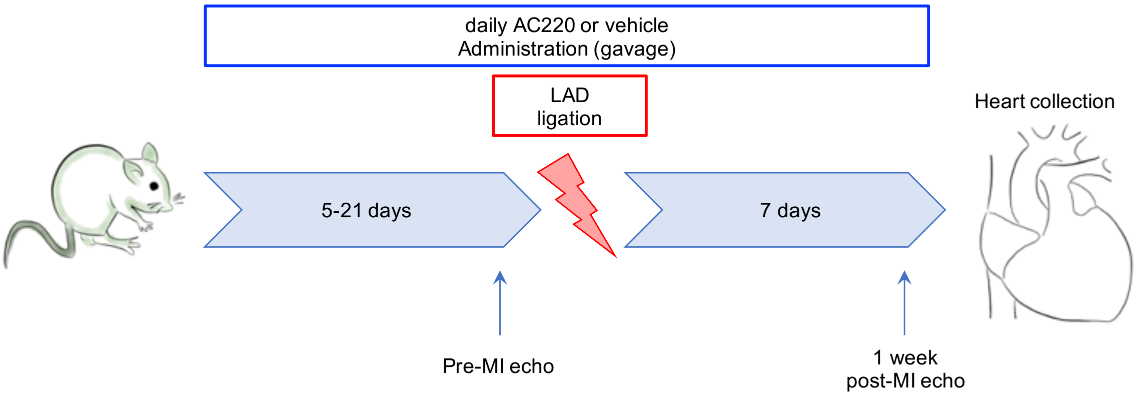

Supplemental Figure S1. Experimental setup. Five to seven-week-old male C57BL/6NRj mice received quizartinib (AC220) or vehicle through oral gavage once a day for a total of 12-28 days. After 5-21 days of treatment animals were randomly assigned to left ventricular anterior descending artery (LAD) ligation or sham operation. One week after surgery mice were sacrificed in deep anesthesia upon completion of echocardiography and hearts were perfused and embedded in paraffin for further immunohistochemistry. Echocardiography was performed before and one week after the surgery.

Supplemental Figure S2

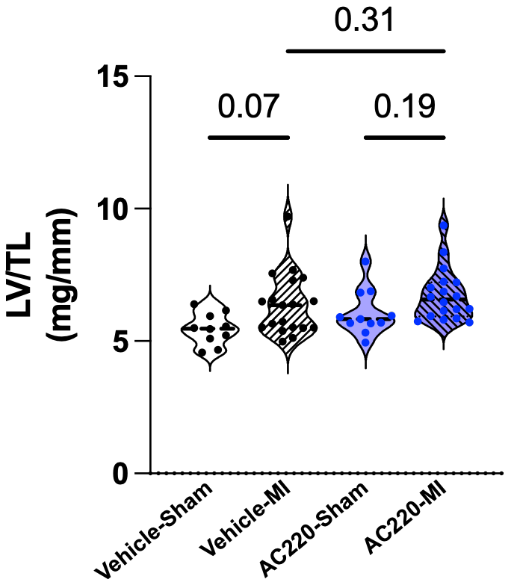

Supplemental Figure S2. Left ventricular weight to tibia length ratio one week after sham or infarct surgery. Post-mortem ratios (mg/mm) of left ventricular (LV) weight to tibia length (TL) in a subset of mice as per Figure 2, in which the hearts were not perfusion-fixed. LV/TL ratios were slightly, but non-significantly higher one week after MI compared to sham surgery, but not different between the respective vehicle and AC220 (quizartinib) groups. Kruskal-Wallis test followed by Dunn's test. LV: left ventricle, TL: tibia length, AC220: quizartinib.

Supplemental Figure S3

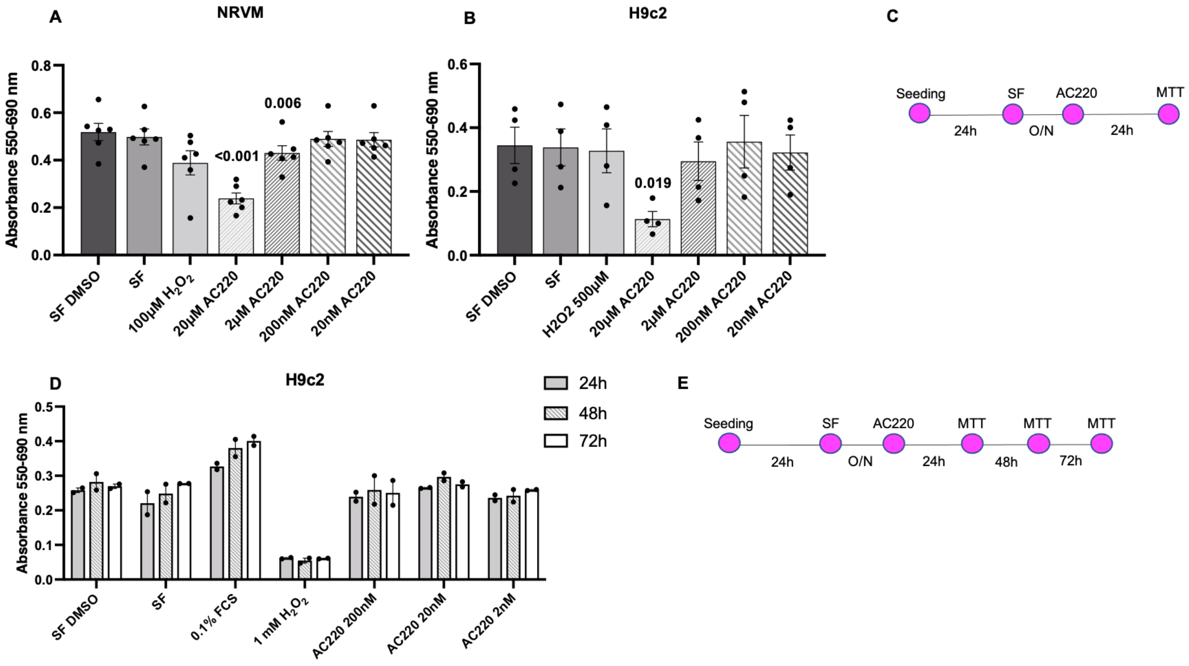

Supplemental Figure S3. Neonatal rat ventricular myocyte (NRVM) and H9c2 cell viability after treatment with different concentrations of quizartinib. MTT assay was performed after 24, 48 and 72 hrs of quizartinib treatment. DMSO, 100 µM, 500 µM and 1 mM H_2O_2 were used as control. In a higher dose-range *in vitro*, quizartinib decreases cardiac myocyte viability, whereas low doses do not have any effect over time. **(A)** Quantification of absorbance in NRVM after 24 hrs, n=6 independent experiments with 6 replicates per each condition; one-way ANOVA followed by Sidak multiple comparisons test. **(B)** Quantification of absorbance in H9c2 cells after 24 hrs, n=4 independent experiments with 6 replicates per each condition; one-way ANOVA followed by Sidak multiple comparisons test. **(C)** Experimental set-up of quizartinib treatment for 24 hrs. **(D)** Quantification of absorbance in H9c2 cells treated with low concentrations of quizartinib up to 72 hrs, n=2 independent experiments with 6 replicates per each condition and time point. **(E)** Experimental set up of low doses quizartinib treatment up to 72 hrs. The data are presented as mean ± SEM. O/N: overnight, SF: serum-free, AC220: quizartinib.

Supplemental Figure S4

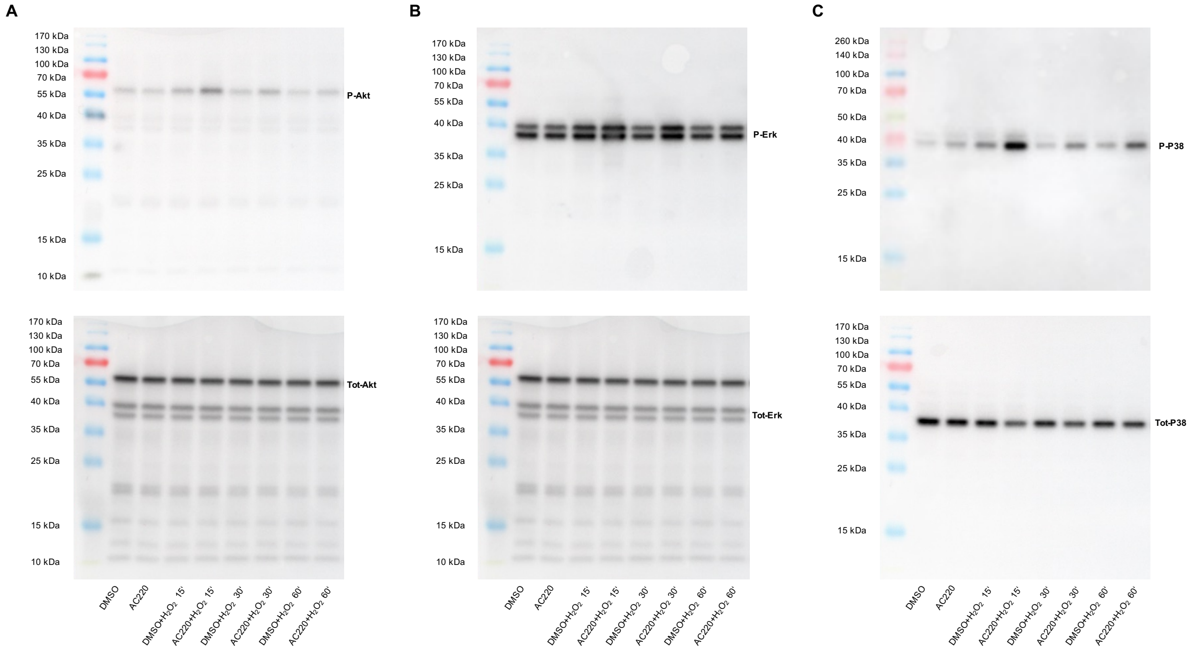

Supplemental Figure S4. Uncropped Western blot images as per Figure 5A-C. **(A)** Uncropped Western blot for phospho-Akt (upper panel) and total Akt (lower panel). **(B)** Uncropped Western blot for phospho-Erk (upper panel) and total Erk (lower panel). **(C)** Uncropped Western blot for phospho-p38 (upper panel) and total p38 (lower panel). Total Akt and total Erk were blotted on the same membrane, the lower panel images of (A) and (B) are therefore identical.
